# Hearing loss and cognitive decline in the general population: a prospective cohort study

**DOI:** 10.1007/s00415-020-10208-8

**Published:** 2020-09-10

**Authors:** Pauline H. Croll, Elisabeth J. Vinke, Nicole M. Armstrong, Silvan Licher, Meike W. Vernooij, Robert J. Baatenburg de Jong, André Goedegebure, M. Arfan Ikram

**Affiliations:** 1 Department of Otorhinolaryngology, Head and Neck Surgery, Erasmus University Medical Center, P.O. Box 2040, 3000 CA Rotterdam, The Netherlands; 2 Department of Epidemiology, Erasmus University Medical Center, Rotterdam, The Netherlands; 3 Department of Radiology and Nuclear Medicine, Erasmus University Medical Center, Rotterdam, The Netherlands; 4 Laboratory of Behavioral Neuroscience, National Institute on Aging, Baltimore, MD USA

**Keywords:** Hearing loss, Presbycusis, Cognitive decline, Aging, Dementia

## Abstract

**Background:**

Previous studies identifying hearing loss as a promising modifiable risk factor for cognitive decline mostly adjusted for baseline age solely. As such a faster cognitive decline at a higher age, which is expected considering the non-linear relationship between cognition and age, may have been overlooked. Therefore it remains uncertain whether effects of hearing loss on cognitive decline extend beyond age-related declines of cognitive function.

**Methods:**

3,590 non-demented participants were eligible for analysis at baseline, and a maximum of 837 participants were eligible for the longitudinal analysis. Hearing loss was defined at baseline. Cognitive function was measured at baseline and at follow-up (4.4 years [SD: 0.2]). Multivariable linear regression analysis was used for the cross-sectional analysis. Linear mixed models were used to assess the longitudinal association between hearing loss and cognitive decline over time while adjusting for confounders and the interaction of age and follow-up time.

**Results:**

Hearing loss was associated with lower cognitive function at baseline. Moreover, hearing loss was associated with accelerated cognitive decline over time on a memory test. After additionally adjusting for the interaction between age and follow-up time, we found that hearing loss did not accelerate cognitive decline anymore.

**Conclusions:**

Hearing loss was associated with lower cognitive function at baseline and accelerated cognitive decline on a memory test. The association between hearing loss and accelerated cognitive decline was non-significant after additional adjustment for non-linear age effects. More evidence is needed to ensure the role of hearing loss as a modifiable risk factor for cognitive decline.

**Electronic supplementary material:**

The online version of this article (10.1007/s00415-020-10208-8) contains supplementary material, which is available to authorized users.

## Introduction

Recently, hearing loss has been put forward as a promising modifiable risk factor for cognitive decline and dementia [5, 28, 29, 32, 45]. Both the prevalence of hearing loss and dementia will increase substantially due to the aging of the worldwide population [2, 15, 32]. With the increasing numbers of both conditions, it is of great importance to determine if hearing loss is independently associated with cognitive decline in dementia-free participants. As such, more can be said on whether hearing rehabilitative treatments may potentially alter or delay the progression of cognitive decline.

Several longitudinal studies reported associations between hearing loss and poorer cognitive function [7], and with an increased risk of dementia [2, 5, 10, 11, 29, 30, 50]. Despite these promising results, several methodological issues should be considered. First, both hearing loss and cognitive impairment are heavily dependent on age, reflected in a steep increase of the prevalence of both with increasing age [15, 20]. Therefore, it is of importance to adjust for both linear and non-linear age effects in the association between hearing loss and cognition. To our knowledge, only one other study incorporated age non-linearly in their models [7]. Yet, it is plausible that older people may decline faster over time on cognitive abilities compared to their younger counterparts. Keeping this age-related decline into account can be accomplished by adding an interaction between age and follow-up time into statistical models. Second, some studies rely on a limited battery of neuropsychological tests for cognitive assessment [9, 12, 13, 16–18, 28, 30, 31, 33, 37, 43, 44]. This potentially increases the likelihood of misclassification of cognitive impairment [22], especially in those with higher levels of hearing impairment. Lower scores on cognitive tests may partially be falsely attributed to cognitive impairment, as individuals might not be able to hear verbal test instructions properly [36, 39]. Third, hearing loss does not necessarily accurately reflect an inability to follow speech in noisy environments [34]. To our knowledge, only one other study incorporated a measure of speech understanding in their analyses [33].

Against this background, we aimed to elucidate whether hearing loss accelerates cognitive decline over time in dementia-free participants whom are at risk of cognitive decline and cognitive impairment from a large population-based study. We measured hearing loss, including speech understanding, and repeatedly assessed cognitive functioning with comprehensive cognitive testing. We examined whether trajectories of cognitive decline differed across degrees of hearing impairment while adjusting for potentially strong effects of age.

## Methods

### Study setting and population

This study is embedded in the Rotterdam Study, a prospective, population-based cohort study. The Rotterdam Study was initiated in 1989 and investigates determinants and consequences of aging. Details of the study have been described previously [21]. The entire study population consists of 14,926 individuals aged ≥ 45 years from the Ommoord area, a suburb of Rotterdam, the Netherlands, who undergo extensive examinations at the research center at study entry and subsequent visits every 3–4 years. In 2011, hearing assessment was introduced into the study protocol. For the present study, we sampled two study populations, described in detail below.

#### Hearing loss and cognitive function: cross-sectional study population

In total, 3739 participants underwent baseline hearing assessment (2011–2014). We excluded participants with probable conductive hearing loss (air–bone gap ≥ 15 dB; *N* = 83), participants with a history of dementia or those who were insufficiently screened for dementia at baseline (*N* = 51), and participants who developed dementia during follow-up (*N* = 15), leaving 3590 participants with baseline hearing assessment. From those 3590 participants, data were available on different cognitive tests, namely the MMSE (*N* = 3584), the Stroop test (*N* = 3500), the Word Fluency test (WFT) (*N* = 3536), the Letter Digit Substitution test (LDST) (*N* = 3507), the Word Learning test (WLT) (*N* = 3239), and the Purdue Pegboard test (PPT) (*N* = 3264). There were 3498 participants with both data on hearing thresholds and speech understanding in noise.

#### Hearing loss and cognitive decline: longitudinal study population

Data on the different cognitive tests from participants who were re-invited for follow-up measurements and with available cognitive data at baseline, were available at follow-up (2015–2016) for the longitudinal analysis. At follow-up, 837 participants had data available for the MMSE, 764 participants for the Stroop test, 519 participants for the WFT, 780 participants for the LDST, 755 participants for the WLT, and 714 participants for the PPT. The mean time interval between cognitive baseline assessment and re-examination was 4.4 years (SD: 0.2). See supplementary methods for an explanation regarding the attrition rate.

### Hearing

#### Hearing thresholds measured with pure-tone audiometry

To determine hearing loss expressed by hearing thresholds in decibel (dB), pure-tone audiometry (PTA) was performed in a soundproof booth [21]. A computer-based audiometry system (Decos Technology Group, version 210.2.6, AudioNigma interface) and TDH-39 headphones were used. dB hearing levels were measured according to the ISO-standard 8253-1 (International Organization for Standardization, 2010). Air conduction (frequencies 0.25–8 kilohertz [kHz]) and bone conduction (0.5 and 4 kHz) were tested for both ears while masking according to the method of Hood [19]. The best hearing ear was determined by taking the average hearing thresholds over all frequencies and identified by the lowest hearing threshold of one of both ears. Of the best hearing ear, we determined the average speech frequencies threshold (average of 0.5, 1, 2, and 4 kHz) levels. Finally, we determined degrees of hearing loss: normal hearing (0–20 dB), mild hearing loss (20–35 dB), moderate hearing loss (35–50 dB), and severe hearing loss (≥ 50 dB) [21, 41].

#### Speech understanding in noise measured with the digits-in-noise test

To measure speech understanding in noise, we derived a signal-to-noise ratio (SNR; in dB) from the digits-in-noise (DIN) test, a 3-min test of speech understanding in noise [25]. Both speech and noise signal were presented in the participant’s better hearing ear. Pre-recorded male-spoken speech-signal consisted of 24 digit triplets. Initially, the triplet was presented at 0 dB SNR. In case of an incorrect response, the next triplet was presented more intensely. After the first correct response, the speech level was decreased and a new stimulus was presented. For a correct response, the intensity was decreased again, while an incorrect response lead to an increase of the intensity. This was repeated until 24 triplets were repeated. SNR was the average of the last 20 triplets. We defined hearing categories based on optimal SNR cut points defined by clinically relevant degree of hearing loss using Youden’s Index (Supplementary Fig. 1) [52].

### Cognition

Cognitive function was assessed in detail with an extensive neuropsychological test battery comprising the MMSE, the Stroop test (adjusted interference score; inverted as higher scores indicate worse performance), the WFT (amount of animals named within 60 s), the LDST (number of correct digits within 60 s), the 15-WLT (total number of words remembered at least 10 min after immediate recall), and the PPT (sum score of three trials). The MMSE was administered during a home visit, the other tests were administered at the research center. All tests instructions were presented verbally. The MMSE is a validated screening tool for cognitive decline and cognitive impairment [46]. The Stroop test is a validated test measuring executive functioning, more specifically it measures the ability to inhibit cognitive interference [40]. To accurately and reliably measure verbal fluency, the WFT was used [42]. With the validated LDST, we measured executive functioning including processing speed and attention [47]. The 15-WLT is a validated test measuring memory functioning [3]. Results of the WLT are not negatively influenced by hearing status, as the 15 different words are visually presented to the participants. The PPT is a validated measure of unilateral and bilateral fine manual dexterity [8].

### Covariates

During home interviews, educational level was assessed and categorized as primary education, lower education, intermediate vocational education and higher education. Smoking habits were assessed during the same interview and subsequently classified into never, former and current smoking [21]. Alcohol consumption was assessed through self-report with the food-frequency questionnaire [48], and we subsequently calculated daily alcohol consumption in grams [48]. Systolic and diastolic blood pressures were measured twice on the right arm with a random-zero sphygmomanometer; the mean of these readings was used for the analyses. Use of antihypertensive medication was assessed by interview [21]. Participants were screened for dementia at baseline and follow-up examinations using a protocol described in detail elsewhere [4].

### Statistics

We investigated whether baseline characteristics differed between participants with just a baseline assessment and participants with both a baseline and a follow-up assessment using *T* tests, *χ*^2^ tests, and Mann–Whitney *U* Tests when appropriate. Subsequently, we present three sequential analyses to examine the association between hearing loss and cognition.

First, we assessed the cross-sectional association between hearing loss (all frequencies, speech frequencies, degrees of hearing loss and SNR) and cognitive functioning at baseline using multivariable linear regression models. We adjusted for age, age^2^, sex, education, alcohol consumption, smoking behavior, systolic- and diastolic blood pressure, and use of blood pressure lowering medication. All SNR analyses were additionally adjusted by PTA hearing levels for all frequencies.

Second, we used linear mixed models with random intercepts and slopes to elucidate the longitudinal association between degrees of hearing loss (mild, moderate or severe compared to normal hearing defined by either PTA or SNR) and cognitive trajectories per test. In each model, we entered follow-up time in years after baseline measurement to use as time variable. For adjustment, we used the same models as described above. In a second model, a two-way interaction between age and follow-up time was added to account for possible slope differences for cognition over time, depending on the baseline age. All SNR analyses were additionally adjusted by PTA hearing thresholds. Next to the linear effects of hearing loss on cognition, an interaction of hearing loss and follow-up time was incorporated in all models, to allow slope differences in the relationship between cognitive functioning and time explained by degree of hearing loss. The linear hearing loss term (intercept difference) and the interaction term between hearing loss and follow-up time (slope difference) are the main terms of interest in this longitudinal analysis. For SNR analysis, random slopes were not included as the models failed to converge.

Third, we performed similar linear mixed models to study the longitudinal association between hearing levels (all frequencies, speech frequencies, and SNR) and cognitive trajectories per test.

In sensitivity analyses, we explored whether longitudinal associations between hearing levels and cognitive trajectories differed between men and women and between mid-life (51–70 years) compared to late life (70–99 years). Originally, the MMSE was designed as a cognitive screening tool and is therefore limited in its capability to truly measure global cognitive functioning [46]. In an additional sensitivity analyses, we created a global cognition score, a *g* factor, by *z* transforming and averaging performance across each of the tests (except for the MMSE). Results were non-significant and effect estimates were smaller than those for the MMSE, indicating that the *g* factor in this sample cannot be considered as a more sensitive marker of global cognition than the MMSE. To facilitate interpretability and comparability (previous studies often used the MMSE), we chose to show the results for the MMSE and omit results regarding the g-factor from the final manuscript.

IBM SPSS Statistics version 25 (International Business Machines Corporation, Armonk, New York) and RStudio; integrated development environment for R, version 3.5.1. (RStudio, Boston, Massachusetts) were used for statistical analyses. Analyses with linear mixed models were done using the “*lme*” function of the R-package “*nlme” *[35]. A *p* value < 0.05 was considered statistically significant.

## Results

Table 1 shows the baseline characteristics of the study population. Mean age was 65.2 years (SD: 7.3). 56.2% of our population were female. Participants had a mean all frequency hearing threshold of 22.8 dB (SD: 11.1). 44.6% of the population had normal hearing threshold levels. For speech understanding in noise, mean SNR was − 4.06 dB (SD: 4.2). Participants with a follow-up assessment compared to participants with only a baseline assessment were significantly older, had a lower alcohol intake and were unhealthier (Supplementary Table 1).

**Table 1:** Baseline characteristics

Baseline characteristics	*N* = 3590
Age [years (SD)]	65.2 (7.3)
Age (range)	51.5–98.6
Female [*N* (%)]	2016 (56.2)
Educational level [*N* (%)]	
Primary	264 (7.4)
Lower	1330 (37.0)
Intermediate vocational	1049 (29.2)
Higher	925 (25.8)
Alcohol consumption^a^, gram (IQR)	7.9 (1.4–19.1)
Smoking [*N* (%)]	
Never	1134 (33.5)
Past	1828 (50.9)
Current	611 (17.0)
Systolic blood pressure, mmHg (SD)	139.5 (21.0)
Diastolic blood pressure, mmHg (SD)	83.1 (11.2)
Use of blood pressure lowering medication [*N* (%)]	1449 (40.4)
Hearing thresholds measured with pure-tone audiometry	
All frequency hearing loss [dB (SD)]	20.8 (9.7)
Speech frequency hearing loss [dB (SD)]	20.0 (10.7)
Degree of hearing loss [*N* (%)]	
Normal (0–20 dB)	1601 (44.6)
Mild (20–35 dB)	1456 (40.6)
Moderate (35–50 dB)	425 (11.8)
Severe (50 dB)	79 (2.2)
Speech understanding in noise measured with the digits-in-noise test	*N* = 3498
Signal-to-noise ratio [dB (SD)]	− 4.06 (4.2)
Degree of hearing loss [*N* (%)]	
Normal (0–20 dB)	1662 (46.3)
Mild (20–35 dB)	837 (23.3)
Moderate/severe (35–50 dB)	1,091 (30.4)
Cognitive abilities	
Mini-Mental State Examination score^a^ (IQR)	29.0 (27.0–29.0)
Stroop Test interference score^a^ (IQR)	44.5 (37.9–54.1)
Word Fluency Test score^a^ (IQR)	23.0 (19.0–27.0)
Letter Digit Substitution Test score^a^ (IQR)	30.0 (26.0–35.0)
Word Learning Test delayed recall score^a^ (IQR)	8.0 (6.0–10.0)
Purdue Pegboard Test sum score^a^ (IQR)	36.0 (33.0–39.0)

Values are mean (standard deviation [SD]) for continuous variables or ^a^ median (interquartile range [IQR]) for non-normally distributed continuous variables and percentages for categorical variables. The amount of hearing loss is expressed in dB, i.e. a higher dB value reflects more hearing loss. Abbreviations: dB, decibel; mmHg, millimetres of mercury

### Cross-sectional results

Table 2 shows the cross-sectional association between hearing loss and cognitive function. Elevated hearing thresholds and diminished speech in noise understanding were associated with lower scores on all cognitive tests, and appeared to be most pronounced for participants with severe hearing loss as compared to normal hearing on the Stroop test, WFT, LDST and the PPT (Table 2).

**Table 2:** Effect estimates of hearing loss and cognitive function based on the cross-sectional analysis

*Hearing loss*	Mini− mental state examination score	Stroop test interference score	Word fluency test score	Letter digit substitution test score	Word learning test delayed recall	Purdue pegboard test sum score
	Difference (95% CI)	Difference (95% CI)	Difference (95% CI)	Difference (95% CI)	Difference (95% CI)	Difference (95% CI)
**Hearing loss measured with pure-tone audiometry**						
*Hearing thresholds per 10 dB increase*						
All frequencies	− 0.04 (− 0.14, 0.06)	− 0.63 (− 1.31, 0.04)	**− 0.42 (− 0.65, − 0.20)**	**− 0.38 (− 0.62, − 0.14)**	**− 0.11 (− 0.23, − 0.00)**	**− 0.33 (− 0.52, − 0.14)**
Speech frequencies	0.01 (− 0.09, 0.11)	− 0.49 (− 1.15, 0.18)	**− 0.37 (− 0.59, − 0.15)**	**− 0.27 (− 0.51, − 0.03)**	− 0.10 (− 0.21, 0.01)	**− 0.28 (− 0.47, − 0.09)**
*Degree of hearing loss*						
Normal (0–20 dB)	Reference	Reference	Reference	Reference	Reference	Reference
Mild (20–35 dB)	− 0.07 (− 0.25, 0.12)	− 0.75 (− 2.18, 0.67)	**− 1.02 (− 1.50, − 0.55)**	− 0.42 (− 0.93, 0.09)	− 0.20 (− 0.44, 0.03)	**− 0.52 (− 0.92, − 0.13)**
Moderate (35–50 dB)	− 0.10 (− 0.44, 0.23)	− 1.84 (− 4.05, 0.37)	**− 0.77 (− 1.50, − 0.03)**	− 0.66 (− 1.45, 0.12)	− 0.31 (− 0.68, 0.05)	**− 0.83 (− 1.45, − 0.21)**
Severe (≥ 50 dB)	**− 0.98 (− 1.94, − 0.02)**	0.02 (− 4.58, 4.61)	**− 1.88 (− 3.40, − 0.37)**	**− 1.91 (− 3.54, − 0.28)**	− 0.59 (− 1.35, 0.16)	**− 1.38 (− 2.67, − 0.09)**
**Hearing loss measured with the digits-in-noise test**						
* Speech understanding in noise per 1 dB increase*						
Speech reception threshold	**− 0.07 (− 0.10, − 0.04)**	**− 0.59 (− 0.96, − 0.23)**	− 0.03 (− 0.12, 0.05)	**− 0.19 (− 0.30, − 0.09)**	**− 0.07 (− 0.12, − 0.02)**	− 0.03 (− 0.06, 0.00)
*Degree of hearing loss*^a^						
Normal (≤ − 5.55 dB)	Reference	Reference	Reference	Reference	Reference	Reference
Mild (− 5.55 to − 3.80 dB)	− 0.14 (− 0.37, 0.09)	− 2,08 (− 4.75, 0.59)	**− 0.80 (− 1.45, − 0.14)**	**− 0.85 (− 1.59, − 0.11)**	**− 0.41 (− 0.77, − 0.05)**	0.01 (− 0.19, 0.22)
Moderate/severe (> − 3.80 dB)	**− 0.36 (− 0.64, − 0.08)**	**− 6.19 (− 9.38, − 2.99)**	**− 0.92 (− 1.71, − 0.14)**	**− 1.56 (− 2.45, − 0.66)**	**− 0.53 (− 0.96, − 0.09)**	**− 0.29 (− 0.54, − 0.04)**

Difference: represents the difference in cognitive score per 10 dB increase in hearing acuity or the difference in cognitive score per 1 dB increase in speech understanding in noise or the difference in degree of hearing loss (both hearing acuity (PTA) and speech understanding (DIN)) as compared to normal hearing. All frequencies: 0.25, 0.50, 1, 2, 4, and 8 kHz. Speech frequencies: 0.5, 1, 2, and 4 kHz. The amount of hearing loss is expressed in dB, i.e. a higher dB value reflects more hearing loss

*CI* confidence interval, *dB* decibel

^a^Defined by digits-in-noise score cut-offs. Adjusted for age, age^2^, sex, education, alcohol consumption, smoking, diastolic and systolic blood pressure, and use of blood pressure lowering medication. Analyses using speech understanding were further adjusted for hearing acuity

Statistically significant effect estimates (*p* < 0.05) are indicated in bold

### Longitudinal results

**Fig. 1:**
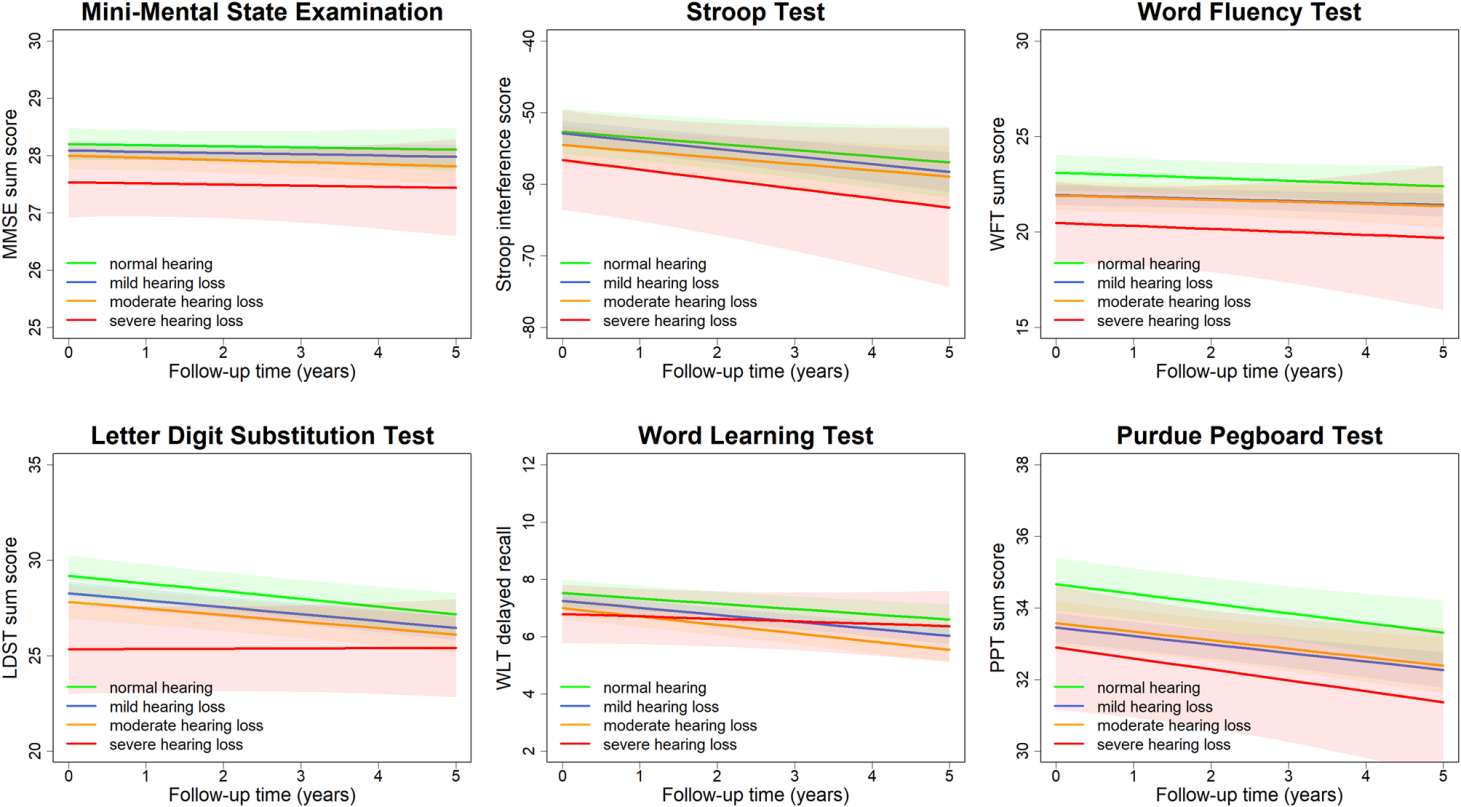
Estimated cognitive function trajectories over time for different degrees of hearing loss as measured with pure-tone audiometry, with corresponding 95% confidence intervals: adjusted for age and sex

In the first model, mild hearing loss showed statistically significant intercept differences, compared to normal hearing thresholds on the WFT, LDST, and the PPT (Table 3). In line with this, mild and moderate degrees of hearing loss, showed intercept differences for all cognitive tests, though not statistically significant (Table 3; model 1). Longitudinally, moderate hearing loss as compared to normal hearing thresholds modified the slope of memory functioning as measured with the 15-WLT significantly over time. For the other cognitive tests no significant slope differences were identified (Table 4; model 1). No significant slope differences were found for any hearing loss, as compared to normal speech understanding in noise (Table 4, model 1). The significant slope difference of the 15-WLT became statistically non-significant, and slope differences of other cognitive tests became small or close to zero (Table 3; model 2; Fig. 1) after additional adjustment for the interaction between age and follow-up time. Comparable results were found for degrees of hearing loss as measured with the DIN test (Table 4; model 2).

**Table 3:** Effect estimates of the degree of hearing loss and cognitive function based on the longitudinal analysis (intercept differences)

*Degree of hearing loss*		Mini-mental state examination score	Stroop test interference score	Word fluency test score	Letter digit substitution test score	Word learning test delayed recall	Purdue pegboard test sum score
		Difference (95% CI)	Difference (95% CI)	Difference (95% CI)	Difference (95% CI)	Difference (95% CI)	Difference (95% CI)
**Degrees of hearing loss as measured with pure-tone audiometry**							
*Normal (0–20 dB)*	Model 1	Reference	Reference	Reference	Reference	Reference	Reference
*Mild (20–35 dB)*	Model 1	− 0.08 (− 0.38, 0.23)	0.11 (− 3.45, 3.67)	− **1.11 (**− **2.19,** − **0.04)**	− 0.64 (− 1.79, 0.51)	− 0.18 (− 0.71, 0.36)	− **1.01 (**− **1.84,** − **0.17)**
*Moderate (35–50 dB)*	Model 1	− 0.14 (− 0.50, 0.21)	− 1.76 (− 5.91, 2.39)	− 1.09 (− 2.34, 0.16)	− 1.07 (− 2.41, 0.27)	− 0.37 (− 0.99, 0.26)	− 0.80 (− 1.77, 0.17)
*Severe (*≥*50 dB)*	Model 1	− 0.33 (− 0.98, 0.32)	− 1.70 (− 9.39, 6.00)	− 2.00 (− 4.33, 0.33)	− 2.42 (− 4.92, 0.07)	− 0.26 (− 1.41, 0.86)	− 0.89 (− 2.79, 1.00)
*Normal (0–20 dB)*	Model 2	Reference	Reference	Reference	Reference	Reference	Reference
*Mild (20–35 dB)*	Model 2	− 0.09 (− 0.39, 0.21)	0.12 (− 3.43, 3.68)	− **1.17 (**− **2.24,** − **0.09)**	− 0.69 (− 1.84, 0.46)	− 0.22 (− 0.75, 0.32)	− **1.03 (**− **1.87,** − **0.20)**
*Moderate (35–50 dB)*	Model 2	− 0.17 (− 0.53, 0.18)	− 1.73 (− 5.88, 2.42)	− 1.23 (− 2.48, 0.03)	− 1.19 (− 2.54, 0.15)	− 0.47 (− 1.10, 0.15)	− 0.87 (− 1.84, 0.11)
*Severe (*≥*50 dB)*	Model 2	− 0.39 (− 1.04, 0.27)	− 1.72 (− 9.41, 5.96)	− 2.24 (− 4.57, 0.10)	− **2.65 (**− **5.16,** − **0.14)**	− 0.46 (− 1.61, 0.69)	− 1.04 (− 2.95, 0.86)
**Degrees of hearing loss as measured with digits-in-noise test**							
*Normal (≤* − *5.55 dB)*	Model 1	Reference	Reference	Reference	Reference	Reference	Reference
*Mild (*− *5.55 to* − *3.80 dB)*	Model 1	− 0.04 (− 0.32, 0.25)	− 1.27 (− 4.53, 1.98)	− 0.88 (− 1.69, − 0.07)	− 0.92 (− 1.83, − 0.01)	− 0.59 (− 1.03, − 0.16)	0.05 (− 0.20, 0.30)
*Moderate/severe (>* − *3.80 dB)*	Model 1	− 0.27 (− 0.60, 0.07)	− 5.30 (− 9.18, − 1.42)	− 1.07 (− 2.04, − 0.11)	− 1.50 (− 2.60, − 0.40)	− 0.73 (− 1.25, − 0.21)	− 0.31 (− 0.60, − 0.02)
*Normal (≤* − *5.55 dB)*	Model 2	Reference	Reference	Reference	Reference	Reference	Reference
*Mild (*− *5.55 to* − *3.80 dB)*	Model 2	− 0.05 (− 0.34, 0.23)	− 1.63 (− 4.87, 1.62)	− 0.92 (− 1.73, − 0.11)	− 0.95 (− 1.86, − 0.03)	− 0.62 (− 1.05, − 0.18)	0.04 (− 0.21, 0.29)
*Moderate/severe (>* − *3.80 dB)*	Model 2	− 0.03 (− 0.64, 0.04)	− 5.96 (− 9.83, − 2.08)	− 1.15 (− 2.12, − 0.19)	− 1.55 (− 2.65, − 0.45)	− 0.77 (− 1.29, − 0.25)	− 0.33 (− 0.62, − 0.03)

Difference: represents the intercept difference in cognitive score per degree hearing loss (both hearing threshold as measured with pure-tone audiometry and speech understanding in noise as measured with the digits-in-noise test) as compared to normal hearing

*CI* confidence interval, *dB* decibel. *Model 1* adjusted for age, sex, education, alcohol consumption, smoking, diastolic and systolic blood pressure, and use of blood pressure lowering medication. *Model 2* additionally adjusted for the interaction between age and follow-up time. Analyses using speech understanding were further adjusted for hearing thresholds as measured with pure-tone audiometry

Statistically significant effect estimates (*p* < 0.05) are indicated in bold

**Table 4:** The additional change in cognitive score per year attributed to different degrees of hearing loss based on the longitudinal analysis (slope differences)

*Degree of hearing loss*		Mini-mental state examination score	Stroop test interference score	Word fluency test score	Letter digit substitution test score	Word learning test delayed recall	Purdue pegboard test sum score
		Difference (95% CI)	Difference (95% CI)	Difference (95% CI)	Difference (95% CI)	Difference (95% CI)	Difference (95% CI)
**Degrees of hearing loss as measured with pure-tone audiometry**							
*Normal (0–20 dB)*	Model 1	Reference	Reference	Reference	Reference	Reference	Reference
*Mild (20–35 dB)*	Model 1	− 0.01 (− 0.10, 0.07)	− 0.13 (− 1.29, 0.43)	− 0.00 (− 0.24, 0.23)	0.01 (− 0.18, 0.21)	− 0.09 (− 0.20, 0.03)	0.02 (− 0.16, 0.19)
*Moderate (35*–*50 dB)*	Model 1	− 0.04 (− 0.14, 0.06)	− 0.47 (− 1.47, 0.52)	− 0.05 (− 0.35, 0.24)	0.00 (− 0.22, 0.23)	− **0.17 (**− **0.30,** − **0.03)**	− 0.01 (− 0.22, 0.19)
*Severe (*≥*50 dB)*	Model 1	− 0.05 (− 0.23, 0.13)	− 1.39 (− 3.20, 0.43)	− 0.19 (− 0.96, 0.58)	0.30 (− 0.14, 0.73)	− 0.03 (− 0.27, 0.22)	− 0.15 (− 0.54, 0.24)
*Normal (0–20 dB)*	Model 2	Reference	Reference	Reference	Reference	Reference	Reference
*Mild (20–35 dB)*	Model 2	− 0.00 (− 0.09, 0.08)	− 0.23 (− 1.09, 0.63)	0.04 (− 0.20, 0.27)	0.04 (− 0.16, 0.24)	− 0.06 (− 0.18, 0.06)	0.03 (− 0.14, 0.21)
*Moderate (35*–*50 dB)*	Model 2	− 0.02 (− 0.12, 0.08)	− 0.03 (− 1.05, 0.98)	0.03 (− 0.28, 0.33)	0.06 (− 0.17, 0.30)	− 0.11 (− 0.25, 0.03)	0.03 (− 0.18, 0.24)
*Severe (*≥*50 dB)*	Model 2	− 0.01 (− 0.19, 0.18)	− 0.47 (− 2.35, 1.41)	− 0.02 (− 0.80, 0.75)	0.42 (− 0.03, 0.88)	0.09 (− 0.17, 0.35)	− 0.05 (− 0.46, 0.36)
**Degrees of hearing loss as measured with the digits-in-noise test**							
*Normal (≤* − *5.55 dB)*	Model 1	Reference	Reference	Reference	Reference	Reference	Reference
*Mild (*− *5.55 to* − *3.80 dB)*	Model 1	− 0.03 (− 0.12, 0.05)	− 0.15 (− 1.01, 0.71)	0.06 (− 0.14, 0.25)	0.04 (− 0.15, 0.24)	0.07 (− 0.04, 0.18)	− 0.04 (− 0.11, 0.03)
*Moderate/severe (>* − *3.80 dB)*	Model 1	− 0.07 (− 0.15, 0.00)	− 0.45 (− 1.20, 0.30)	0.04 (− 0.14, 0.21)	− 0.04 (− 0.21, 0.13)	0.04 (− 0.06, 0.13)	− 0.02 (− 0.09, 0.04)
*Normal (≤*− *5.55 dB)*	Model 2	Reference	Reference	Reference	Reference	Reference	Reference
*Mild (*− *5.55 to* − *3.80 dB)*	Model 2	− 0.03 (− 0.11, 0.06)	0.06 (− 0.79, 0.91)	0.08 (− 0.12, 0.27)	0.06 (− 0.14, 0.25)	0.08 (− 0.03, 0.19)	− 0.03 (− 0.11, 0.04)
*Moderate/severe (>* − *3.80 dB)*	Model 2	− 0.04 (− 0.12, 0.03)	0.10 (− 0.66, 0.87)	0.10 (− 0.08, 0.28)	0.00 (− 0.17, 0.18)	0.07 (− 0.03, 0.17)	− 0.01 (− 0.08, 0.05)

Difference: represents the additional change in cognitive score per year increase in follow-up time per degree hearing loss (both hearing threshold as measured with pure-tone audiometry and speech understanding in noise as measured with the digits-in-noise test) as compared to normal hearing

*CI* confidence interval. dB: decibel. *Model 1* adjusted for age, sex, education, alcohol consumption, smoking, diastolic and systolic blood pressure, and use of blood pressure lowering medication. *Model 2* additionally adjusted for the interaction between age and follow-up time. Analyses using speech understanding were further adjusted for hearing thresholds as measured with pure-tone audiometry

Statistically significant effect estimates (*p* < 0.05) are indicated in bold

Moreover, assessing hearing levels continuously showed that the additional change in cognitive functioning attributable to hearing loss were small and non-significant for both hearing thresholds and speech understanding in noise (Supplementary Table 2). Results did not differ between males and females or between midlife and late-life (Supplementary Tables 3 and 4).

## Discussion

In this large population-based study in non-demented older adults at risk for cognitive decline and cognitive impairment, we found that hearing loss was associated with poorer global cognitive functioning, executive functioning, verbal fluency, attention, memory, and manual dexterity. After adjustment for the possible non-linear effects of age on cognitive change during follow-up, we did not find that hearing loss for either hearing thresholds or speech understanding in noise accelerates cognitive decline over time.

Strengths of this study are its prospective and longitudinal population-based design, the large sample size and the standardized assessment of hearing thresholds with pure-tone audiometry and a speech-in-noise test as well as cognitive functioning with comprehensive cognitive testing. However, the following limitations of this study must be considered. First, although we extensively adjusted for age and other important confounders, residual confounding might still be present. For example, frailty and psychosocial well-being may confound our results as those are known to be highly related to age-related hearing loss [1, 51]. Second, dementia incidence of participants with a baseline hearing assessment is small (*N* = 15), precluding the possibility to analyze whether hearing loss is associated with an increased risk of dementia in this sample.

Our cross-sectional results were comparable with other studies, reflected in lower scores on cognitive tests with higher levels of hearing loss [14, 28, 37]. It is of great interest that based on our results hearing loss (both peripheral and central) seems to affect executive functioning, verbal fluency, memory, manual dexterity and to some extent global cognitive functioning. Previous studies have argued that hearing loss leads to an increased cognitive load, shifting cognitive capacities towards sensory impairments rather than cognition [50]. Therefore, processes such as attention, memory, executive functioning, inhibitory control and verbal fluency may be compromised as a result of hearing loss. Moreover, cognitive decline, and especially diminished executive functioning, has repeatedly been linked to an increased risk of general frailty [38]. Even though we cannot infer on causality in this cross-sectional analysis, it does shed important light on a general risk of frailty in elderly with hearing impairment and comorbid compromised cognitive functioning. This underlines the great importance of investigating whether timely rehabilitative hearing treatment may alter or delay cognitive decline and possibly lowering the risk of full-blown dementia and/or general frailty [6, 51].

In our longitudinal analysis we found an accelerated decline in memory function (as measured with the 15-WLT) with moderate hearing loss, which is comparable to the results and effect estimates of other population-based studies [7, 30]. Unexpectedly, we did not find such an association for participants with severe levels of hearing loss, which could be explained by its relatively low prevalence (2.2%). Importantly, with further adjustment for confounding by age, the association between hearing loss and memory function became weaker and statistically non-significant. The prevalence of both hearing- and cognitive impairment increases substantially with age [15, 32]. Moreover, it is also important to consider, especially in longitudinal studies with a wider age range that older individuals may decline faster on cognitive test performance between baseline and follow-up measurement than their younger counterparts [36]. Therefore, we added the interaction between baseline age and follow-up time into our statistical models, which seemed to explain most of the effects of hearing loss on memory function as the slope difference becomes statistically non-significant in the second model. To our knowledge, only one other study incorporated non-linear effects of age in their statistical model [7]. Therefore, verification in future studies is needed to explore whether effects of hearing loss on cognitive decline extend beyond ‘normal’ age-related decline of cognitive function.

Besides elevated hearing thresholds, speech understanding in noise could contribute towards accelerated cognitive decline. The ability to understand speech in noise is a complex process in which elements of peripheral processing interact with more centrally located elements of auditory processing [25]. As such, it may be hypothesized that a potential association with cognitive functioning may even be stronger when specifically speech understanding is reduced. Interestingly, we found the same (non-significant) results between speech understanding in noise and cognitive decline. This may be explained by the fact that there is a high correlation between hearing loss based on audiometry and speech in noise results in our population [25].

It is also worthwhile considering whether found associations in our and previous studies might be driven by confounding and/or bias. The absence of an effect of hearing loss on cognitive decline in the current study is not explained by selection bias, as the sample with both a baseline- and a follow-up measurement were significantly older than the participants with just a baseline measurement. Moreover, it has been proposed that upstream common causes, i.e., inflammation, vascular pathology, and other systemic neurodegenerative processes, may lead to both hearing loss and cognitive decline through central nervous system-wide functional decline, rather than that those two are directly related to one another [36]. As such, greater sensitivity in one domain could identify impairments in that domain prior to the other, leading to the appearance of a false direct association [36, 39].

We should also acknowledge that our follow-up time (mean = 4.4 years) may have been too short to capture a possible small, but significant effect of hearing loss on cognition. Epidemiological evidence has shown that elevated blood pressure in mid-life, an established modifiable risk factor of dementia, increases the risk of cognitive impairment 20–30 years later [23, 24, 26, 49]. In contrast, another study with a follow-up of 8 years did not find an association between hypertension and cognitive functioning [27]. The differences in these results suggest that the follow-up time would need to be longer to show a potential association of hearing loss with cognitive decline. Nevertheless, despite the relatively short follow-up time, we do find an effect of mild hearing loss on memory functioning in the first model which is both statistically significant as well as clinically relevant [5, 7]. Would our follow-up time truly been too short to capture an effect of hearing loss on cognitive function, we would have expected non-significant results.

In conclusion, hearing loss was significantly associated with compromised cognitive function and with accelerated decline on the 15-WLT measuring memory function. Notably, the latter association seemed to be driven by non-linear effects of age. Future, population-based studies are needed to further confirm the role of hearing loss as a potential modifiable risk factor for cognitive decline, whilst paying attention to effects of age on cognition. Even though more research is needed to strengthen evidence between hearing loss and accelerated neurodegeneration, our results do underline the great importance to acknowledge the effects of hearing loss (whether it being direct or indirect) on compromised cognitive function and associated general frailty within the elderly.

## Electronic supplementary material

Below is the link to the electronic supplementary material.

Supplementary file1 (PDF 185 kb)

## Data Availability

Data can be obtained on request. Requests should be directed toward the management team of the Rotterdam Study (secretariat.epi@erasmusmc.nl), which has a protocol for approving data requests. Because of restrictions based on privacy regulations and informed consent of the participants, data cannot be made freely available in a public repository.

## Code availability

Code can be obtained on request which should be directed to the corresponding author.
