# Supplementary material for: Hearing loss and cognitive decline in the general population: a prospective cohort study
Source: J Neurol. 2020 Sep 10;268(3):860–71. doi: 10.1007/s00415-020-10208-8 (PMC7914236; doi:10.1007/s00415-020-10208-8)
Supplement: Supplementary file 1 — Supplementary file1 (PDF 185 kb) [file 415_2020_10208_MOESM1_ESM.pdf]

SUPPLEMENTARY FILE

SUPPLEMENTARY METHODS

Clarification attrition rate between baseline- and follow-up measurement

Supplementary figure 2 shows a schematic overview of the Rotterdam Study. The Rotterdam Study consists of four different cohorts: RS-I (from 1990 onwards), RS-II (from 2000 onwards), RS-III (from 2006 onwards), and RS-IV (from 2007 onwards). As hearing assessment was incorporated into the core study protocol from 2011 onwards, data on baseline hearing function has been collected in RS-I (halfway through visit 5), RS-II (visit 3), and RS-III (visit 2). Data of RS-IV is not available yet and is thus not included in the current study. Of the cohorts with available baseline data, RS-I (visit 6) and RS-II (visit 4) have had a follow-up assessment. RS-III, which contains 2,504 participants with a baseline hearing- and cognitive assessment, has not been re-invited to the study center yet for follow-up examinations.

SUPPLEMENTARY DATA

Supplementary table 1. Demographic differences

**Baseline characteristics**	Participants with only baseline cognitive measurement	Participants with both baseline and FU cognitive measurements	Significance
Age, years	63.0 (6.6)	71.9 (4.4)	**
Female, %	57.0	53.7	n.s.
Education level, %			
Primary	8.4	4.2	**
Lower	35.9	41.6	**
Intermediate vocational	28.5	32.8	**
Higher	27.2	21.9	**
Alcohol consumption, gram	8.1 (1.4 – 20.0)	6.9 (1.2 – 17.0)	*
Smoking, %			
Never	31.8	31.6	n.s.
Past	18.8	11.8	**
Current	49.4	56.6	**
Systolic blood pressure, mmHg	135.8 (19.6)	151.0 (20.9)	**
Diastolic blood pressure, mmHg	82.1 (10.9)	86.3 (11.4)	**
Use of blood pressure lowering medication, %	37.6	49.4	**

Values are mean (standard deviation) for continuous variables or median (interquartile range) when indicated ([a]), percentages for dichotomous variables. T-test were used for normally distributed variables, χ^2-test for dichotomous variables, and Mann-Whitney U-Test for non-normally distributed variables to see whether characteristics were significantly different ($p < 0.05$) between participants with only a baseline cognitive assessment and participants with a second cognitive assessment at follow-up. Statistically significant difference between groups: * $p < 0.05$. ** $p < 0.01$.

Supplementary table 2. The additional change in cognitive score per year attributed to hearing loss based on the longitudinal analysis (slope differences)

Hearing loss		**Mini-Mental State Examination score**	**Stroop Test interference score**	**Word Fluency Test score**	**Letter Digit Substitution Test score**	**Word Learning Test delayed recall**	**Purdue Pegboard Test sum score**
		Difference (95% CI)	Difference (95% CI)	Difference (95% CI)	Difference (95% CI)	Difference (95% CI)	Difference (95% CI)
Hearing threshold per 10 dB increase							
All frequencies	Model 1	-0.01 (-0.04, 0.02)	-0.12 (-0.42, 0.18)	-0.02 (-0.11, 0.08)	0.02 (-0.05, 0.08)	-0.03 (-0.07, 0.01)	0.01 (-0.05, 0.07)
	Model 2	0.00 (-0.03, 0.03)	0.09 (-0.21, 0.41)	0.02 (-0.08, 0.12)	0.05 (-0.02, 0.12)	-0.01 (-0.05, 0.03)	0.03 (-0.04, 0.09)
Speech frequencies	Model 1	-0.02 (-0.05, 0.01)	-0.12 (-0.41, 0.18)	-0.03 (-0.12, 0.07)	0.00 (-0.06, 0.07)	-0.03 (-0.07, 0.01)	-0.01 (-0.07, 0.05)
	Model 2	-0.01 (-0.04, 0.02)	0.07 (-0.24, 0.37)	0.00 (-0.10, 0.10)	0.03 (-0.04, 0.10)	-0.01 (-0.05, 0.03)	0.00 (-0.06, 0.07)
Speech understanding in noise per 1 dB increase							
Signal-to-noise ratio	Model 1	-0.01 (-0.02, 0.00)	-0.06 (-0.13, 0.02)	0.00 (-0.02, 0.01)	0.00 (-0.01, 0.02)	0.00 (-0.01, 0.01)	0.00 (0.00, 0.01)
	Model 2	-0.01 (-0.02, 0.00)	-0.02 (-0.09, 0.06)	0.00 (-0.02, 0.02)	0.00 (-0.01, 0.02)	0.00 (-0.01, 0.01)	0.00 (0.00, 0.01)

Difference: represents the additional change in cognitive score per year increase in follow-up time per 10 dB increase in hearing threshold as measured with pure-tone audiometry or the additional change in cognitive score per year increase in follow-up time per 1 dB increase in speech reception threshold as measured with the digits-in-noise test. All frequencies is the average of: 0.25, 0.50, 1, 2, 4, and 8 kHz. Speech frequencies is the average of: 0.5, 1, 2, and 4 kHz. The amount of hearing loss is expressed in dB, i.e. a higher dB value reflects more hearing loss. CI: confidence interval. dB: decibel. Model 1: adjusted for age, sex, education, alcohol consumption, smoking, diastolic and systolic blood pressure, and use of blood pressure lowering medication. Model 2: additionally adjusted for the interaction between age and follow-up time. Analyses for speech understanding were further adjusted for hearing thresholds as measured with pure-tone audiometry. Statistically significant effect estimates ($p < 0.05$) are indicated in **bold**.

Supplementary table 3. The longitudinal association between hearing loss and cognitive decline stratified on sex

		Mini-Mental State Examination score	Stroop Test interference score	Word Fluency Test score	Letter Digit Substitution Test score	Word Learning Test delayed recall	Purdue Pegboard Test sum score
		Difference (95% CI)	Difference (95% CI)	Difference (95% CI)	Difference (95% CI)	Difference (95% CI)	Difference (95% CI)
	**Male**						
Hearing threshold	***All frequencies***	-0.01 (-0.09, 0.06)	-0.01 (-0.37, 0.35)	0.02 (-0.14, 0.17)	0.08 (-0.03, 0.19)	0.00 (-0.06, 0.06)	-0.03 (-0.13, 0.06)
	Speech frequencies	-0.04 (-0.11, 0.03)	-0.08 (-0.43, 0.27)	-0.00 (-0.16, 0.15)	0.06 (-0.04, 0.16)	0.00 (-0.06, 0.06)	-0.06 (-0.15, 0.03)
Speech understanding	***Signal-to-noise ratio***	-0.01 (-0.02, 0.01)	-0.05 (-0.17, 0.07)	0.00 (-0.03, 0.03)	0.02 (-0.01, 0.05)	0.00 (-0.02, 0.02)	0.00 (-0.01, 0.01)
	**Female**						
Hearing threshold	***All frequencies***	-0.04 (-0.09, 0.02)	0.18 (-0.22, 0.59)	0.04 (-0.09, 0.17)	0.02 (-0.08, 0.12)	-0.02 (-0.07, 0.04)	**0.09 (0.00, 0.18)**
	Speech frequencies	-0.05 (-0.10, 0.01)	0.13 (-0.28, 0.53)	0.02 (-0.11, 0.15)	0.00 (-0.10, 0.10)	-0.03 (-0.08, 0.02)	0.06 (-0.02, 0.15)
Speech understanding	***Signal-to-noise ratio***	-0.01 (-0.02, 0.00)	-0.01 (-0.10, 0.09)	0.00 (-0.03, 0.02)	-0.01 (-0.03, 0.01)	0.00 (-0.01, 0.01)	0.00 (0.00, 0.01)

Difference: represents the additional change in cognitive score per year increase in follow-up time per 10 dB increase in hearing threshold as measured with pure-tone audiometry or the additional change in cognitive score per year increase in follow-up time per 1 dB increase in speech-reception threshold as measured with the digits-in-noise test. All frequencies is the average of: 0.25, 0.50, 1, 2, 4, and 8 kHz. Speech frequencies is the average of: 0.5, 1, 2, and 4 kHz. The amount of hearing loss is expressed in dB, i.e. a higher dB value reflects more hearing loss. CI: confidence interval. dB: decibel. Adjusted for age, sex, education, alcohol consumption, smoking, diastolic and systolic blood pressure, use of blood pressure lowering medication, and for the interaction between age and follow-up time. Analyses with speech understanding were further adjusted for hearing thresholds as measured with pure-tone audiometry. Statistically significant effect estimates ($p < 0.05$) are indicated in **bold**.

Supplementary table 4. The longitudinal association between peripheral hearing loss and cognitive decline stratified on midlife versus late-life

		Mini-Mental State Examination score	Stroop Test interference score	Word Fluency Test score	Letter Digit Substitution Test score	Word Learning Test delayed recall	Purdue Pegboard Test sum score
		Difference (95% CI)	Difference (95% CI)	Difference (95% CI)	Difference (95% CI)	Difference (95% CI)	Difference (95% CI)
	**Midlife (51 – 70 years)**						
Hearing threshold	***All frequencies***	0.01 (-0.06, 0.07)	-0.09 (-0.46, 0.28)	-0.06 (-0.21, 0.09)	0.03 (-0.08, 0.14)	0.01 (-0.05, 0.08)	0.05 (-0.05, 0.16)
	Speech frequencies	-0.01 (-0.07, 0.06)	-0.17 (-0.54, 0.19)	-0.08 (-0.24, 0.08)	-0.00 (-0.11, 0.11)	0.01 (-0.06, 0.07)	0.04 (-0.06, 0.15)
Speech understanding	***Signal-to-noise ratio***	0.00 (-0.01, 0.01)	-0.01 (-0.08, 0.07)	0.00 (-0.03, 0.03)	0.01 (-0.02, 0.04)	0.01 (-0.01, 0.03)	0.00 (-0.01, 0.01)
	**Late-life (70 – 99 years)**						
Hearing threshold	***All frequencies***	-0.05 (-0.11, 0.01)	0.30 (-0.15, 0.75)	0.04 (-0.09, 0.18)	0.07 (-0.03, 0.17)	-0.02 (-0.08, 0.03)	0.01 (-0.08, 0.09)
	Speech frequencies	-0.06 (-0.12, 0.00)	0.27 (-0.16, 0.70)	0.03 (-0.10, 0.16)	0.06 (-0.04, 0.15)	-0.03 (-0.08, 0.03)	-0.03 (-0.11, 0.06)
Speech understanding	***Signal-to-noise ratio***	-0.01 (-0.02, 0.00)	-0.03 (-0.15, 0.08)	0.00 (-0.03, 0.02)	-0.01 (-0.03, 0.02)	0.00 (-0.01, 0.01)	0.00 (-0.01, 0.01)

Difference: represents the additional change in cognitive score per year increase in follow-up time per 10 dB increase in hearing threshold as measured with pure-tone audiometry or the additional difference in cognitive score per year increase in follow-up time per 1 dB increase in speech-reception threshold as measured with the digits-in-noise test. All frequencies is the average of: 0.25, 0.50, 1, 2, 4, and 8 kHz. Speech frequencies is the average of: 0.5, 1, 2, and 4 kHz. The amount of hearing loss is expressed in dB, i.e. a higher dB value reflects more hearing loss. CI: confidence interval. Adjusted for age, sex, education, alcohol consumption, smoking, diastolic and systolic blood pressure, use of blood pressure lowering medication, and for the interaction between age and follow-up time. Analyses with speech understanding were further adjusted for hearing thresholds as measured with pure-tone audiometry. Statistically significant effect estimates ($p < 0.05$) are indicated in **bold**.

Supplementary figure 1. Receiver operating curves illustrating the optimal cut-points for both mild hearing loss and moderate or greater hearing loss, as compared to normal hearing.

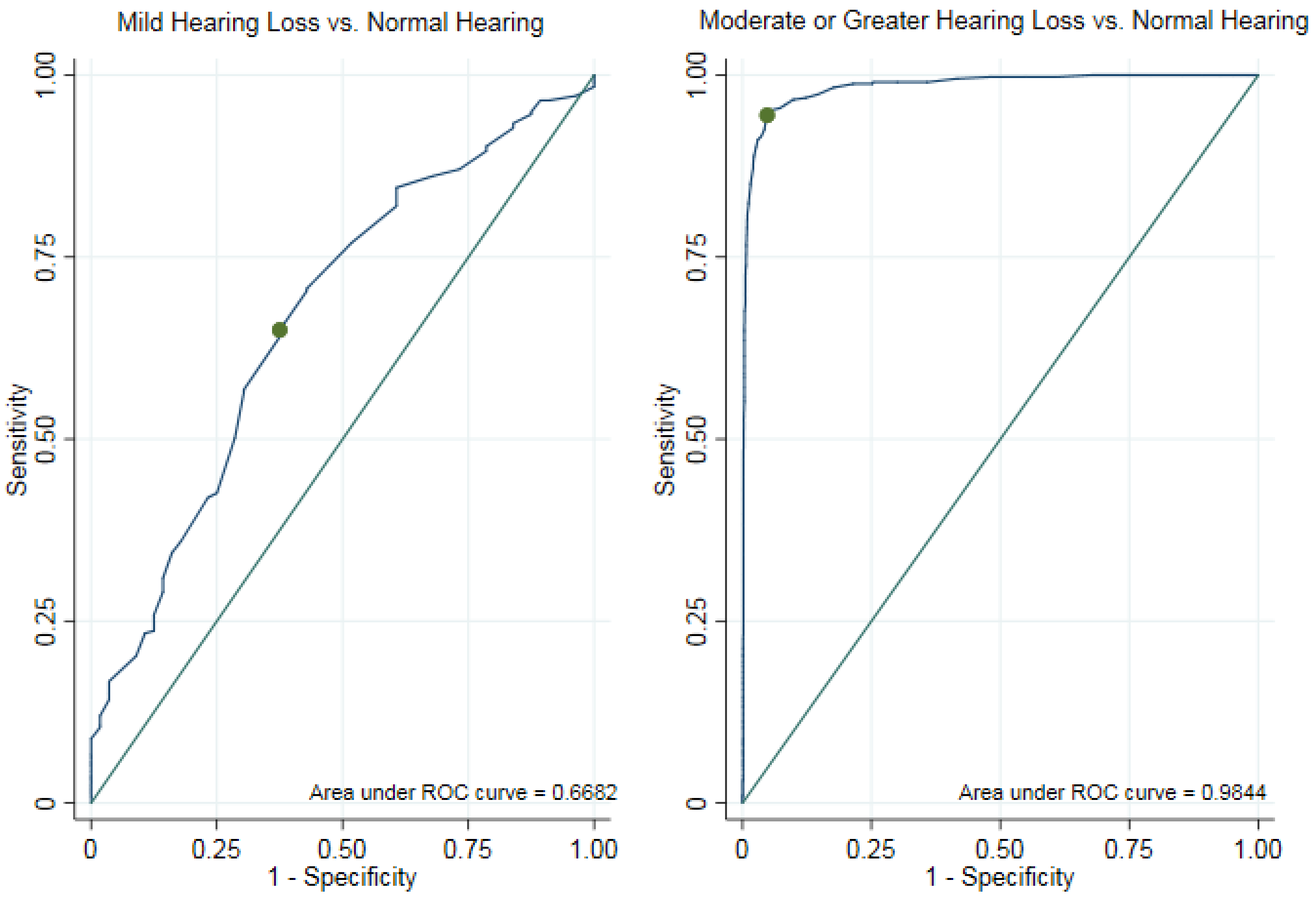

Receiver operating curves show the optimal cut-points for both mild hearing loss and moderate or greater hearing loss, based on pure-tone averages. The degrees of hearing loss defined by pure-tone average are: normal hearing (0 – 20 dB), mild hearing loss (20 – 35 dB), and moderate or greater hearing loss (>35 dB). This also shows the area under the ROC curve, a measure of how well the speech recognition levels from the Digits-In-Noise test can distinguish between the hearing groups.

Supplementary figure 2. Schematic overview of the Rotterdam Study.

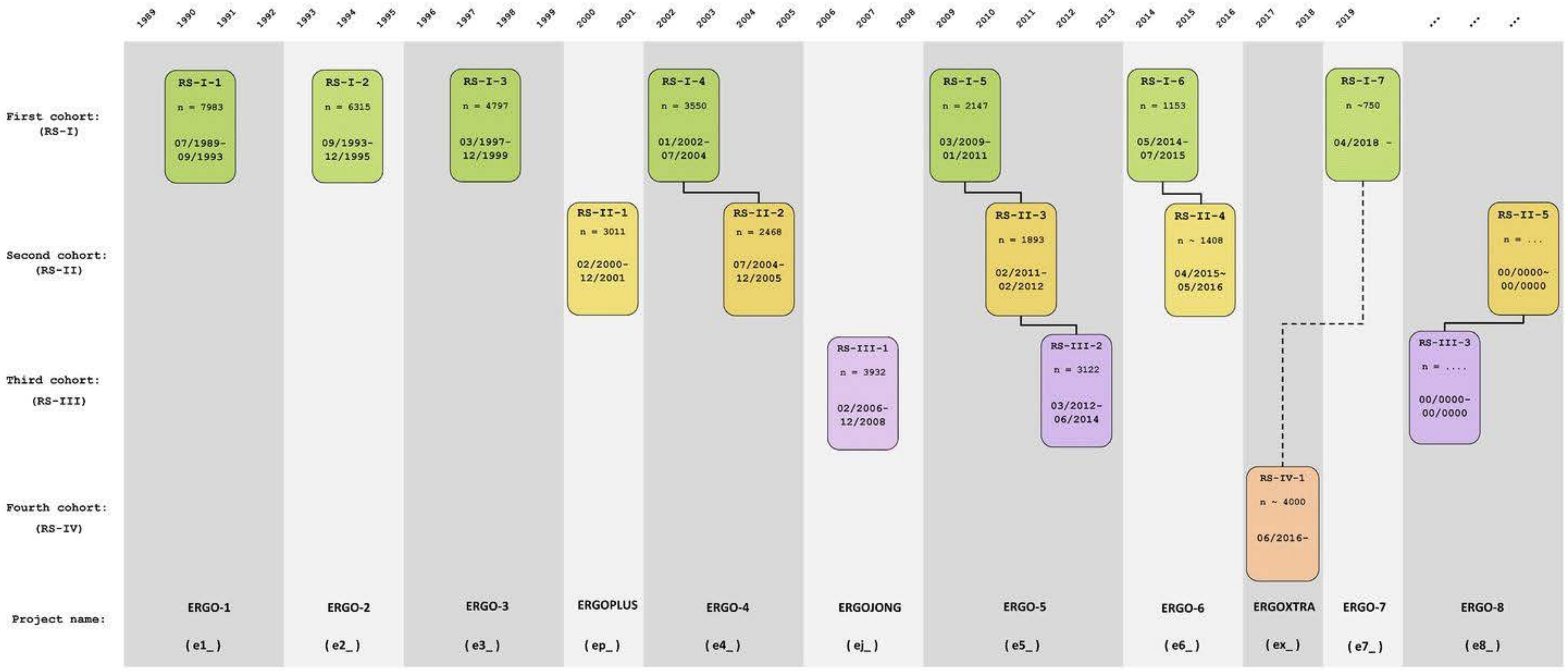

Supplementary figure 2 shows a schematic overview of the cohorts of the Rotterdam Study. Hearing assessment was added to the core study protocol in 2011; halfway through RS-I-5, at the start of RS-II-3 and RS-III-2. Data of RS-IV is under embargo and thus not available yet.
